# Quantitative Analysis of Size‐Dependent Structural Disorder in Ruthenium Nanoparticles by Crystal PDF Full‐Space Refinement

**DOI:** 10.1002/smtd.202501982

**Published:** 2026-04-20

**Authors:** Satoshi Hiroi, Hirotaka Ashitani, Okkyun Seo, Jaemyung Kim, Chulho Song, Loku Singgapulige Rosantha Kumara, Chen Yanna, Koji Ohara, Kohei Kusada, Hiroshi Kitagawa, Osami Sakata

**Affiliations:** ^1^ Faculty of Materials for Energy Shimane University Matsue Shimane Japan; ^2^ Japan Synchrotron Radiation Research Institute Hyogo Japan; ^3^ RIKEN SPring‐8 Center Hyogo Japan; ^4^ NISSAN ARC, LTD. Yokosuka Kanagawa Japan; ^5^ Canadian Light Source 4 Innovation Boulevard Saskatoon SK Canada; ^6^ The Hakubi Center for Advanced Research Kyoto University Kitashirakawa Oiwakecho Kyoto Japan; ^7^ Institute for Advanced Study (IAS) Kyushu University Fukuoka Japan; ^8^ Faculty of Engineering Sciences Kyushu University Fukuoka Japan; ^9^ Graduate School of Science Kyoto University Kitashirakawa Oiwakecho Kyoto Japan

**Keywords:** crystal PDF full‐space refinement, extended unit cell, pair distribution function, point defects, stacking fault

## Abstract

Ruthenium (Ru) nanoparticles (NPs) have attracted significant attention because of their high catalytic activity, which is closely tied to their atomic‐scale structural disorder. In this study, we investigate size‐dependent structural disorders in both face‐centered cubic (fcc) and hexagonal close‐packed (hcp) Ru NPs by pair‐distribution‐function (PDF)‐based structural refinement, called crystal PDF full‐space refinement. High‐energy X‐ray total scattering experiments were conducted, and the experimental PDFs were analyzed using an extended unit cell (EUC) model to quantify stacking faults. The results reveal that all Ru NPs, irrespective of their nominal phase, exhibit mixed stacking sequences of fcc and hcp layers. A strong correlation between NP size and stacking fraction was found in fcc‐based NPs, whereas hcp‐based particles exhibited prominent asymmetry in short‐range PDFs, consistent with a higher density of point‐defect‐like coordination losses. The presence of such defects was supported by peak intensity reduction and lattice contraction trends. Although a direct relationship between point defects and catalytic activity remains unclear, our findings underscore the importance of local atomic arrangements in determining the functionality of Ru NPs. This study demonstrates the utility of crystal PDF full‐space refinement for revealing hidden disorders in nanocrystalline systems and lays the groundwork for rational catalyst design through structural control.

## Graphical abstract

A crystal PDF full‐space refinement approach integrates Bragg diffraction and total scattering to evaluate stacking faults and PDF degradation caused by point defects in ruthenium nanoparticles. This method unveils distinct size‐dependent stacking evolution in fcc‐ and hcp‐based systems, providing a unified and quantitative approach for nanoscale structural refinement.

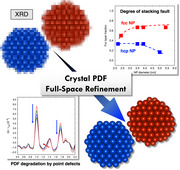

## 1 Introduction

Catalytic materials have been developed for decades and have become indispensable for large‐scale chemical synthesis. Catalysts are also installed in automobiles, where they purify toxic gases such as nitrogen oxides (NO*_x_*) and carbon monoxide (CO) generated by the combustion of fossil fuels. Without such catalysts for toxic gas purification, air pollution would accelerate markedly, causing serious harm to ecosystems. Therefore, the development of catalysts with high purification performance is of great interest and significant societal importance. However, catalysts often contain large amounts of precious metals, making their production costly. Furthermore, owing to recent political instability, the prices of such metals have been rising [1]. To ensure a sustainable supply of widely used catalysts, there is a pressing need to develop alternative materials that are both more cost‐effective and exhibit high catalytic activity.

Over the past decades, nanoparticles (NPs) have garnered significant attention in the development of various functional materials. NPs, typically defined as particles with diameters less than 10 nanometers, exhibit exceptionally large surface areas. Generally, NPs are expected to exhibit enhanced catalytic activity because catalytic reactions occur near the surface. Kitagawa and colleagues have developed and reported NPs of various elements using chemical reduction methods [2, 3, 4, 5, 6, 7, 8]. Notably, they have successfully synthesized crystallographically interesting Ru NPs [9]. Ru does not form a face‐centered cubic (fcc) structure in its bulk metal because a hexagonal close‐packed (hcp) structure is more stable. Surprisingly, Kitagawa and colleagues synthesized fcc‐based Ru NPs over a decade ago and demonstrated that they exhibit superior catalytic performance for CO oxidation compared with hcp‐based NPs.

Understanding the origin of catalytic activity in NPs depends on accurately describing their atomic arrangements. NPs generally do not exhibit sufficient periodicity in the atomic configuration owing to the spatial limitation. This makes nanoscale structural characterization complicated because of the broadened Bragg peaks and the strong diffuse scattering [10]. Scanning transmission electron microscopy (STEM) analysis is one of the most powerful methods of directly observing the atomic configuration. Previous NP studies by STEM have successfully demonstrated differences in stacking sequences between fcc and hcp structures [6, 7, 9]. However, owing to the spatial limitations of STEM, it cannot provide structural information that represents the entire sample, thus requiring averaging multiple STEM images to determine the whole structural trend. In contrast, the atomic pair distribution function (PDF) describes the atomic pair correlation strength in real space. It is particularly suitable for the structural analysis of finite‐sized materials such as NPs because PDF detects atomic pairs in short‐range regions regardless of structural periodicity [11, 12, 13]. Moreover, PDF measurement employs the same optical setup as that in X‐ray diffraction, essentially providing structural information corresponding to the entire sample. Consequently, the structural analysis based on PDF can provide overall structural trends in NP samples, overcoming limitations associated with STEM and conventional diffraction‐based analyses.

To elucidate the origin of the high catalytic performance of fcc‐based Ru NPs, structural analyses have been conducted employing reverse Monte Carlo (RMC) modeling [14, 15, 16, 17] and Rietveld refinement. The Rietveld analysis of Ru NPs conducted by Song et al. determined basic crystal structural variables such as lattice constants and temperature factors by refining fcc and hcp structures [18]. Subsequently, the results of the nonperiodic RMC modeling of Ru NPs were reported by Kumara et al. [19]. Moreover, their results of the analysis of three‐dimensional atomic configurations confirmed ABCABC and ABABAB stacking in both fcc‐based and hcp‐based Ru NPs. Therefore, it was presumed that the presence of stacking faults should be considered when describing the atomic configuration of Ru NPs. Seo et al. reported their Rietveld analysis of fcc‐based Ru NPs, taking stacking faults into account on the basis of the above presumption [20]. As revealed in the literature, stacking faults exist in fcc‐based Ru NPs; furthermore, the probability of finding a stacking fault increases only in the smallest NPs. However, no Rietveld‐based analysis taking into account stacking faults in more stable hcp‐based Ru NPs has been reported to date. The stronger diffuse scattering from hcp‐based NPs than from fcc‐based NPs makes it challenging to evaluate the precise intensity of Bragg peak profiles [19]. Therefore, the application of Rietveld analysis or conventional methods to hcp‐based NPs may include significant errors. To comprehensively elucidate the structural features of hcp‐based Ru NP catalysts, a structural refinement considering stacking faults, which is similarly applied to fcc‐based NPs, is essential.

In 2020, Hiroi et al. developed a structural refinement technique, crystal PDF full‐space refinement utilizing the PDF derived from high‐energy X‐ray diffraction [21]. This method has demonstrated exceptional suitability for materials exhibiting structural disorders [22, 23, 24, 25]. NPs, owing to their finite periodicity, exhibit broader Bragg peaks than bulk metals. Furthermore, the presence of structural defects leads to intense diffuse scattering alongside the Bragg peaks. The PDF includes structural information of both Bragg peaks and diffuse scattering. Although the average structural information derived from Bragg peaks is uniformly distributed through the PDF, structural information from diffuse scattering is typically localized to short‐range regions (*r* < 10 Å). Consequently, it is possible to simply extract the average structural information by referring to the long‐range portion of the PDF. Additionally, since the PDF in short‐range regions corresponds to coordination numbers of neighboring atoms, variations in point defect concentrations in crystals and NPs can be qualitatively evaluated.

In this study, we aim to quantify stacking faults and point defects in Ru NPs by crystal PDF full‐space refinement to clarify their size‐dependent structural features relevant for catalysis. Here, crystal PDF full‐space refinement is conducted on fcc‐ and hcp‐based Ru NPs. The results of the refinement are utilized to elucidate stacking faults and other localized strain phenomena in the Ru NPs. Furthermore, the effect of point defects present in the Ru NPs, as described by the PDF, is investigated. We establish a PDF‐based methodological framework that quantifies stacking sequence and size/defect descriptors in Ru NPs. We discuss qualitative implications for catalysis and how these structural features may influence activity; however, establishing quantitative structure–activity correlations is beyond the scope of the present study.

## 2 Experimental Procedure

Ru NPs were synthesized as described by Kusada et al. [9]. The Ru NPs were characterized as having fcc‐based and hcp‐based structures from their X‐ray diffraction profiles. The average diameters of the NPs and the abbreviations are summarized in Table 1. Representative low‐magnification TEM images of Ru NPs prepared by the same synthetic protocol are available in Kusada et al. [9]. The NP powders were loaded in a 2‐mm inner‐diameter quartz capillary. X‐ray total scattering measurements of the sample powders were performed at the high‐energy X‐ray diffraction beamline BL04B2 in SPring‐8 (Hyogo, Japan) [26]. The incident X‐ray beam with an energy of 61.4 keV was monochromated by the Si 220 reflection of a bent monochromator installed in the beamline. The X‐ray photons scattered from the sample powders were counted using four CdTe detectors (X‐123CdTe, Amptek) and two highly pure Ge solid‐state detectors (GL0515, CANBERRA Industries), which were installed every 8°. The measurements were performed at 2*θ* from 0.3° to 49°; hence, the maximum momentum transfer *Q*_max_ was approximately 25 Å^–^^1^. The coherent X‐ray scattering intensity *I*(*Q*) was obtained by subtracting the X‐ray absorption, the Compton scattering, and the contribution of the quartz capillary and a pure poly(vinylpyrrolidone) (PVP) from the raw X‐ray scattering intensity. The structure factor *S*(*Q*) was obtained by normalizing *I*(*Q*):

(1) $$S\;\left(Q\right)=\frac{I\left(Q\right)-\langle {\left|f\left(Q\right)\right|}^{2}\rangle }{{\left|\langle f\left(Q\right)\rangle \right|}^{2}}+1$$

where $\left\langle {\left|f(Q)\right|}^{2}\right\rangle =\sum_{j}c_{j}|f_{j}\left(Q\right){|}^{2}$ and $\left\langle f(Q)\right\rangle =\sum_{j}c_{j}f_{j}\left(Q\right)$. *c_j_* and *f_j_* (*Q*) are the mole fraction and the atomic form factor of the *j‐*th element in the material, respectively. The PDF, *G*(*r*), was calculated by the Fourier transform of *S*(*Q*):

(2) $$G\;\left(r\right)=\frac{2}{\pi }\int Q\left[S\left(Q\right)-1\right]\sin\mathrm{Qr}dQ$$

**TABLE 1:** Average diameters of fcc‐ and hcp‐based NPs.

Structure	Abbreviation	Diameter (nm)
FCC‐based NP [9]	fcc24	2.4 ± 0.5
	fcc35	3.5 ± 0.7
	fcc39	3.9 ± 0.8
	fcc54	5.4 ± 1.1
HCP‐based NP [9]	hcp22	2.2 ± 0.5
	hcp35	3.5 ± 0.6
	hcp39	3.9 ± 0.6
	hcp50	5.0 ± 0.7

The upper limit of the integral was *Q*_max_, which was determined by the experimental conditions. The analyses were sequentially executed using the original software for the BL04B2 beamline [26].

### 2.1 Statistical Analysis

#### 2.1.1 Pre‐Processing of Data

High‐energy X‐ray total scattering patterns were corrected for background subtraction, including the PVP reference, and absorption/polarization corrections, normalized by atomic form factor to obtain the structure factors *S*(*Q*), and Fourier‐transformed with a Lorch modification. Unless noted, *Q*_min_​ = 0.3 Å^–^^1^, *Q*_max_​ = 25 Å^–^^1^, Δ*Q* = [0.01] Å^–^^1^; *G*(*r*) was evaluated for *r* = 0.8–60 Å with Δ*r* = 0.01 Å.

#### 2.1.2 Data Presentation

Values are mean ± SD unless otherwise stated. *n* denotes independent measurements (distinct specimens/beamtime frames aggregated per specimen before statistics).

#### 2.1.3 Model Fitting and Uncertainty

Crystal PDF full‐space refinement (unit cell‐based EUC) was performed using the nonlinear least squares method. We report *R*_p_​, refined lattice parameters, the atomic displacement parameters, and the crystallite size/strain terms.

Pair‐function fits for the short‐range region used non‐linear least squares for peak position, coordination number, and *σ* confidence intervals are profile‐likelihood CIs; when peaks overlap strongly, bounds were regularized by shared‐width constraints.

#### 2.1.4 Simulation‐To‐Experiment Matching

Debye finite‐cluster simulations (sphere/rod/disk; aspect = 0.6, 0.8, 1.0, 1.4, 1.8; fcc/hcp/EUC model; vacancy = 0, 2, 4, 6, 8%) were post‐processed identically to the experiment. For lognormal size distributions, both number‐weighted and volume‐weighted (*d* ^3^) ensemble averages were computed; The *d* ^3^ weighting emphasizes the larger tail of the distribution and is reported because diffracted intensity scales with total scatterers per particle.

#### 2.1.5 Software

diffpy.srreal 1.4.0, diffpy.structure 3.3.1 for Debye simulations. An in‐house C code for crystal PDF full‐space refinement. Igor Pro macros at SPring‐8 BL04B2 to obtain the *S*(*Q*) and *G*(*r*).

## 3 Analysis

### 3.1 Crystal PDF Full‐Space Refinement

A crystal PDF full‐space refinement of crystalline materials was performed using the self‐made software developed by Hiroi and coworkers [21, 22, 23, 24, 25] to investigate the atomic structure of samples. The coherent scattering intensity from a crystalline phase, *I*_calc_(*Q*), was calculated as

(3) $$I_{\mathrm{calc}}\;\left(Q\right)=\frac{2\pi^{2}}{\mathrm{NV}}\sum_{\tau }\frac{{\left|F\left(\tau \right)\right|}^{2}}{{\left|\tau \right|}^{2}}R\left(Q-\;\left|\tau \right|\right)$$

where *N* is the number of atoms in the structural model with the volume *V* and *R*(*Q*—|τ|) is the *Q*‐broadening function [27, 28]. The *Q*‐broadening function is determined using the instrumental resolution function and the crystallographic domain size or the diameter of NPs. *F*(***τ***) is the crystal structure factor on the reciprocal lattice point **τ**:

(4) $$F\;\left(Q\right)=\sum_{j}^{N}f_{j}\left(\left|Q\right|\right)\exp\left(iQ\cdot r_{j}\right)$$

where **Q** is the reciprocal lattice vector, *f_j_* (|**Q**|) is the atomic scattering factor of the *j*‐th atomic site, and **r** *_j_* is the atomic position. The structure factor and PDF of the crystalline phase, *S*_calc_(*Q*) and *G*_calc_(*r*), can be obtained by calculating Equations 1 and 2 using Equation 3. Average structural parameters of the crystal, i.e., lattice constants, domain size, and the mean square displacement of atoms, were refined by fitting *G*(*r*) in the region of 2.0–60 Å. The fitting region covers the interatomic neighboring distance (2.7 Å) and the maximum Ru NP size (54 Å). In this study, the reliability factor *R*_p_, which is minimized during refinement, was calculated as:

(5) $$R_{p}=\sqrt{\frac{\sum_{j}{\left\{G^{\exp}\left(r_{j}\right)-G^{\mathrm{calc}}\left(r_{j}\right)\right\}}^{2}}{\sum_{j}{\left\{G^{\exp}\left(r_{j}\right)\right\}}^{2}}}\;.$$

The crystal PDF full‐space refinement method refines crystal structure parameters in the same manner as the Rietveld method [29, 30]. The difference between them lies in the fitting process, which is performed using a nonlinear least squares method with PDF data rather than diffraction intensities [21]. Note that Bragg peaks, which are observed in the large wavenumber region, vanish at room temperature. Therefore, the typical range for Rietveld analysis is *Q* < 10 Å^–^^1^. Conversely, in crystal PDF full‐space refinement, the entire wavenumber region can be used because both Bragg peaks and diffuse scattering are analyzed. This enables the analysis of atomic defects and thermal vibrations of atoms, which appear as diffuse scattering due to the high spatial resolution of the PDF. In addition, the Bragg peaks of NPs are markedly broadened owing to their very small correlation length corresponding to NP diameters. The broadened Bragg peaks and diffuse scattering overlap, making it difficult to refine the structural parameters by the Rietveld method. In contrast, the PDF can separate these contributions on the basis of the difference in correlation length. crystal PDF full‐space refinement can determine the parameters with high accuracy owing to the features of the PDF [21, 23]. The crystal PDF full‐space refinement employs the PDF, the same as PDFgui [31, 32], for the calculations. The essential difference between the structural refinement and PDFgui lies in the inclusion of scattering intensity calculations in the reciprocal space. Consequently, the crystal PDF full‐space refinement naturally enables the evaluation of the profile function, the instrumental function derived from optics, and the crystallographic domain size, similarly to Rietveld analysis. In this study, the crystallite size, the strain in crystallites, the isotropic atomic displacement parameter, the lattice constant, and the profile function based on the asymmetric pseudo‐Voigt function [33] are employed as refinement variables.

### 3.2 Extended Unit Cell Model

**FIGURE 1:**
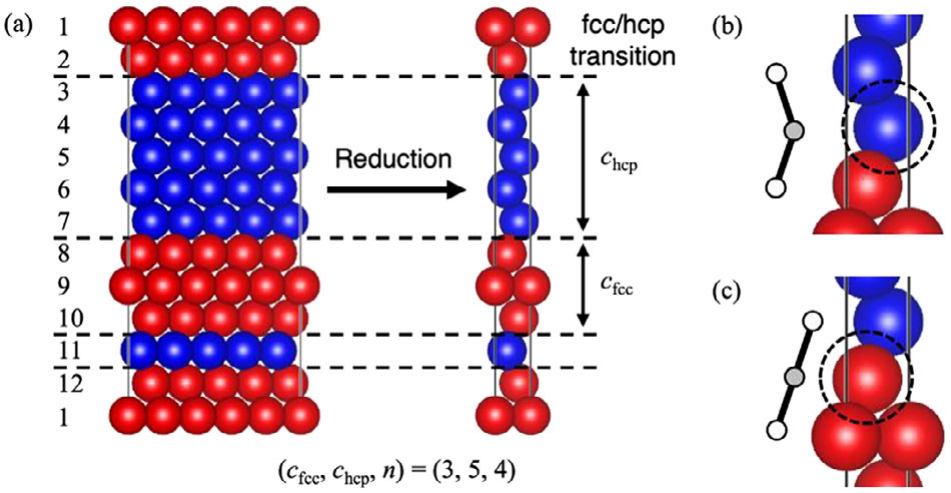
(a) Schematics of the EUC model in Ru NP. Red balls for fcc layer and blue balls for hcp layer. The three parameters correspond to the characterizing parameters for EUC (*c*_fcc_, *c*_hcp_, *n*) (see Table S1). (b) Definition of hcp layer. (c) Definition of fcc layer.

Ashitani et al. introduced the extended unit cell (EUC) model with the aim of representing the stacking faults in the atomic arrangement of PdPt NPs [34]. In this study, the crystal lattice with the 111 orientation of the fcc crystal was defined as the c‐axis of the standard structure of the EUC, and the length of the *c*‐axis was defined as 12 layers. The definition of hcp stacking was given with the in‐plane coordinates of the upper and lower layers of the specified layer being the same, and that of fcc stacking was given with those being not the same. To identify the many possible EUC models, they were characterized by three variables. The maximum number of consecutive fcc layers in a periodic EUC was defined as *c*_fcc_, and the maximum number of consecutive hcp layers was defined as *c*_hcp_. The number of times the fcc/hcp layer transition occurred was defined as *n*. The schematics of the EUC model in a Ru NP are shown in Figure 1. In this study, structural refinement calculations were performed for the 33 EUC models shown in Table S1.

In the structural analysis based on the EUC model, Rietveld analysis is generally used for refinement because it is a crystal structural analysis method based on the unit cell. Consequently, refinement is conducted using the correlation length of a crystal lattice. In contrast, PDFgui directly calculates the correlation length of atomic pairs. Therefore, it is necessary to refine virtual atomic clusters of the same size as the maximum value of the PDF. The *c*‐axis length of the EUC model employed in this study is approximately 26 Å, which implies that even the fcc54 structure can only accommodate two periods. This constraint raised concerns about potential issues during refinement. The crystal PDF full‐space refinement used in this study, similar to Rietveld analysis, is a crystallographic analysis method based on the unit cell. Consequently, structural refinement using the EUC model can be performed using the same procedure as in Rietveld analysis.

### 3.3 Finite‐Cluster Debye Simulation

To quantify the effects of finite size, shape, and defects without assuming long‐range periodicity, the scattering intensity *I*(*Q*) of finite Ru clusters was computed via the Debye scattering equation [35] $I\;\left(Q\right)=\sum_{i}\sum_{j}f_{i}\left(Q\right)f_{j}\left(Q\right)\frac{\sin(Qr_{ij})}{Qr_{ij}}$. Atomistic clusters were built from fcc, hcp, and EUC stacking models, particle shapes (sphere/rod/disk), and aspect ratios were imposed geometrically on a large supercell, and point‐vacancy fractions were introduced by random site removal. To estimate the effect of the size distribution, 300 NP diameters were sampled according to a log‐normal distribution, and both number‐weighted and *d* ^3^ (volume‐weighted) ensemble averages of *I*(*Q*) were computed. The Debye summation and subsequent PDF reduction were implemented using DiffPy.srreal v1.4.0 (DebyePDFCalculator) [36].

From *I*(*Q*), we removed the self‐scattering term and normalized the data to obtain *S*(*Q*), applied a Gaussian *Q*‐damping to mimic instrumental resolution. Then, the *G*(r) was evaluated by Equation (2) with a Lorch window in the Fourier transform​. The same *Q*‐range and reduction steps were used as in the experiment to enable comparisons.

Scattering‐relevant weighting was discussed using the NP size distribution. Since diffracted intensity scales with the number of scatterers per particle, both number‐weighted and *d* ^3^ (volume)‐weighted log‐normal ensembles were compared; the latter is the natural weighting for diffraction. The calculation conditions for the size distribution are summarized in Table S4.

## 4 Result and Discussion

**FIGURE 2:**
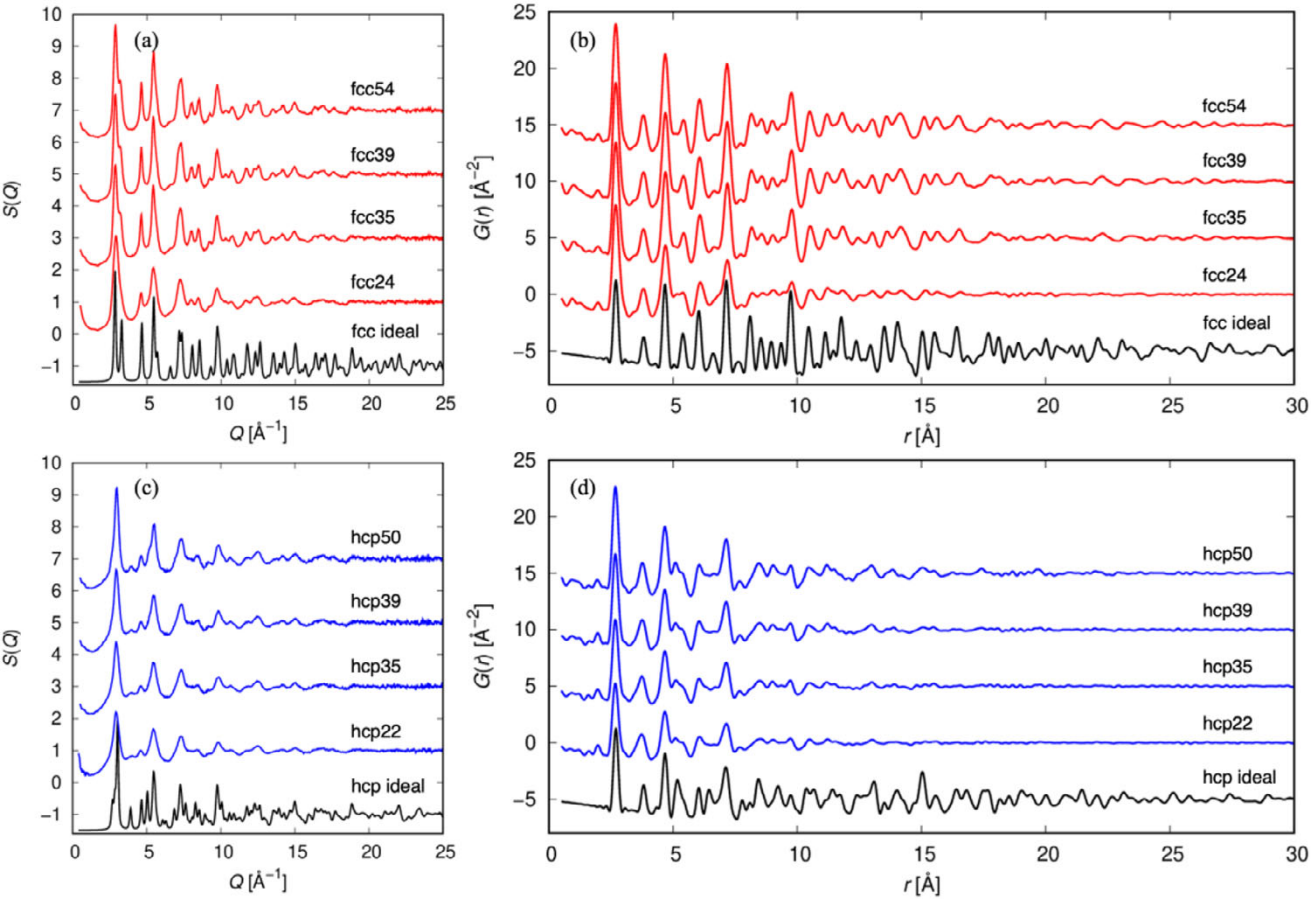
(a, c) Experimental *S*(*Q*) of Ru NPs with fcc and hcp structures. (b, d) Experimental *G*(*r*) of Ru NPs with fcc and hcp structures.

Figure 2 presents the structure factors and PDFs of Ru NPs obtained by X‐ray total scattering measurements. The structure factors of the fcc‐based and hcp‐based NPs exhibit similarities to those of ideal fcc and hcp structures, respectively. The diffuse scattering in the structure factors of the NPs indicates the presence of disorders in the atomic coordinates. The shape of the Bragg peaks in the structure factors tends to broaden as the NP size diminishes. This broadening is attributed to the same reason as the decrease in crystallite size; the range within which the NPs can be considered single crystals corresponds to the diameter of the NPs. In the short‐range region, the experimental *G*(*r*) of the NPs aligns well with the calculated *G*(*r*) of ideal fcc and hcp structures. However, the comparison with the ideal structure becomes challenging because of the rapid decay of *G*(*r*) after 8–10 Å.

**FIGURE 3:**
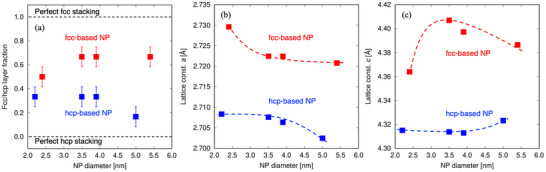
NP diameter dependence of (a) fcc layer fraction, (b) lattice constant *a*, and (c) lattice constant *c* calculated by PDF‐based structural refinement. The color of points indicates the fcc‐ (red) and hcp‐based (blue) NPs. The lattice constant *c* corresponds to the thickness of two fcc/hcp layers. The red and blue broken lines are guides to the eye.

**FIGURE 4:**
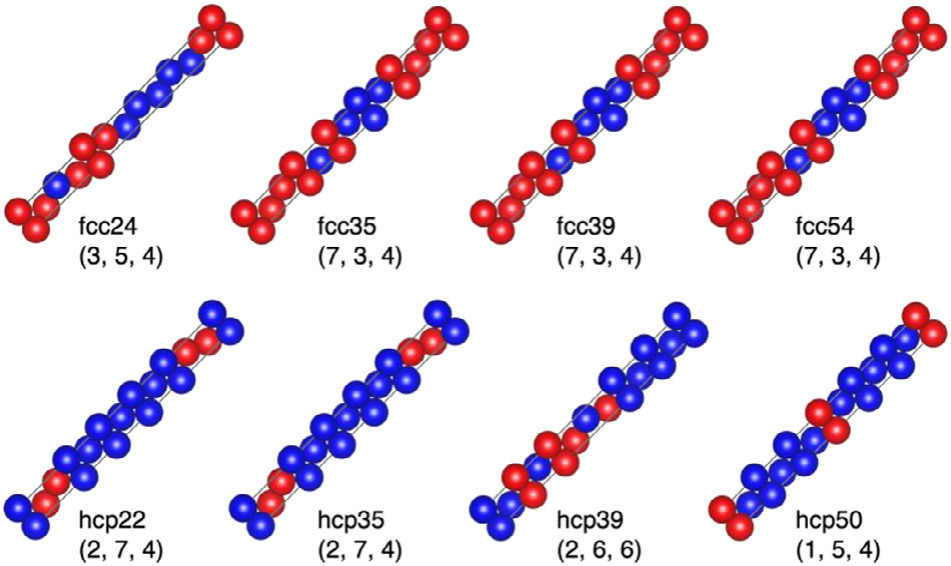
EUC structural models for the best fit to PDFs of each Ru NP. The three parameters correspond to the characterizing parameters for EUC (*c*_fcc_, *c*_hcp_, *n*).

The stacking faults in the NPs were analyzed by crystal PDF full‐space refinement using the EUC model. The refined structural parameters are summarized in Table S2. Figure 3a shows the NP size dependence of the fcc layer fraction of the EUC model with the smallest *R*_p_ obtained by the structural refinement (see Figure S1). These refined EUC models are illustrated in Figure 4 together with their characterizing parameters. The fcc layer fraction takes only seven values, namely, 0, 1/6, 2/6, …, 1, as shown in Table S1. Notably, the fcc layer fraction of all Ru NPs is neither 0 nor 1. This implies that both fcc‐ and hcp‐based NPs are composed of crystal structures mixed with fcc and hcp layers. In fcc‐based NPs, the fcc layer fraction is 4/6 for fcc35, fcc39, and fcc54, whereas it is only 3/6 for fcc24. Conversely, in hcp‐based NPs, only hcp50 exhibits a fraction of 1/6, whereas hcp22, hcp35, and hcp39 have a fraction of 2/6. These results support the correspondence between the fcc layer fraction and the Bragg peak profile. Interestingly, despite the identical number of fcc and hcp layers in fcc24, the Bragg peak profile of fcc24 closely resembles that of fcc. According to the Rietveld analysis results of Seo et al. [20], the probability of finding a stacking fault, *α*, was 0.48 for fcc24, and for the other fcc35, fcc39, and fcc54, *α* was approximately 0.24–0.26. The agreement between the fcc layer fraction and *α* indicates the validity of the crystal PDF full‐space refinement employing the EUC model. The variations in lattice constants of the EUC crystal structures obtained by the structural refinement are shown in Figure 3b, c. Note that the *c*‐axis length corresponds to the thickness of two fcc and hcp layers. The lattice constants exhibited a correlation with the fcc layer fraction. In fcc‐based NPs, which have a large fcc layer fraction, both the lattice constants *a* and *c* are larger than those in hcp‐based NPs. Consequently, the calculated lattice constants suggest that fcc‐ and hcp‐based NPs exhibit distinct structural features. The lattice constants of fcc24 exhibit a distinct trend compared with those of other fcc‐ and hcp‐based NPs. Notably, the lattice constant *a* is the largest among all samples, whereas a shortening in the *c*‐axis direction is observed in fcc24 relative to other fcc‐based NPs. Therefore, this result suggests the formation of a unique layer compressed along the *c*‐axis in fcc24.

Finite‐cluster Debye simulations clarify the role and the limitations of the EUC description at sizes of a few nanometers. For pure fcc/hcp clusters, reducing the particle size produces the expected uniform Bragg broadening and a monotonic damping of *G*(*r*). However, when the EUC model drawn in Figure 1 is implemented as a finite, truncated layer stack, the peak decay becomes non‐uniform (see Figure S8). This reflects an intrinsic termination effect of truncating the stacking sequence and should not be conflated with changes in the fcc/hcp layer fraction. Consequently, within crystal PDF full‐space refinement based on unit cell structure, the EUC model is used as a descriptor of stacking statistics whose fit sensitivity is predominantly governed by the fcc‐layer fraction, whereas finite size and shape effects are addressed by the Debye equation. The choice of ensemble weighting produces predictable but modest differences: *d* ^3^‐weighted averaging enhances the contribution of the largest particles in a distribution, resulting in slightly slower real‐space damping and narrower reciprocal‐space peaks. This effect is most visible for mean diameters at 2 nm and becomes negligible above 4–5 nm (see Figure S13). Throughout the manuscript, when quoting a single mean size for comparison with scattering, we use the volume‐weighted mean. Consistent with the rod– and disk–like morphologies of hcp Ru [9], the refined effective crystallite size tends to be smaller for hcp than for fcc at comparable mean diameters (Table S2). Finite‐cluster Debye simulation with anisotropic masks reproduces the stronger low‐*Q*/long‐*r* damping than the isotropic configuration due to a reduced coherence length.

Table 2 shows the minimum values of *R*_p_ obtained from the structural refinement for each Ru NP. For fcc‐based NPs, there was an agreement, with the exception of fcc24. This result indicates that each EUC model can adequately describe the stacking fault of fcc‐based NPs. However, since the *R*_p_ of the fcc24 NPs did not decrease sufficiently, it can be inferred that there exist local disorders or other defects that the EUC model cannot adequately account for. The *R*_p_ of the hcp‐based NPs was significantly lower than that of the fcc‐based NPs. *R*_p_ is dependent on the NP size, and for hcp22, *R*_p_ reached 20.29%. This result indicates that hcp‐based NPs contain defects beyond stacking faults, and that the number of these defects tends to increase as the NP size decreases.

**TABLE 2:** Minimum *R_p_* values in various EUC models for each NP.

Structure	NP1	NP2	NP3	NP4
fcc NP	fcc24	fcc35	fcc39	fcc54
*R*_p_	16.29%	11.94%	11.27%	11.76%
hcp NP	hcp22	hcp35	hcp39	hcp50
*R*_p_	20.29%	19.62%	17.67%	15.96%

Figure S2 shows a comparison of the experimental and calculated *S*(*Q*) obtained from the EUC minimizing *R*_p_. For the fcc‐based NPs, these *S*(*Q*) correspond to the entire *Q* range. Conversely, for the hcp‐based NPs, a good agreement between these values was observed with the exception of the Bragg peak located approximately 4 Å^–^^1^, which is characteristic of the hcp structure. Therefore, the fcc layer fraction of the hcp22, hcp35, and hcp39 NPs may be smaller than those obtained in this study. The comparison of the experimental and calculated *G*(*r*) shown in Figure S3 demonstrates a good agreement for all the NPs. However, there exists a discrepancy between the experimental and calculated *G*(*r*) around the second peak at 3.7 Å for the hcp‐based NPs. Figure S4 shows a comparison of the cumulative *R*_p_ (*R*_pc_(*r*)) as a function of the distance *r*. The *R*_pc_(*r*) of hcp‐based NPs increased rapidly at around 3.7 Å. In hcp22, hcp35, and hcp39, 35%–45% of *R*_p_ arose from this discrepancy. High *R*_p_ values indicate that stacking faults alone cannot explain hcp‐based NPs, suggesting point defects. A comparison of the experimental and calculated *G*(*r*) reveals that the second peak of the experimental *G*(*r*) extends toward the short‐distance side. Notably, the first, second, and third peaks of *G*(*r*) are observed in all EUC models, which have the most closely packed atomic arrangement (see Figure S5). As long as the EUC model is assumed, the asymmetric second peak cannot be adequately described. Consequently, this asymmetry suggests the existence of additional defects, such as point defects.

**FIGURE 5:**
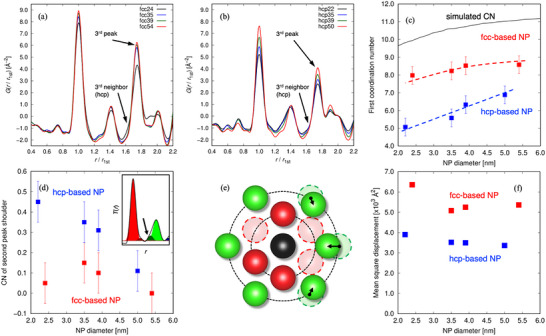
(a, b) Enlarged experimental *G*(*r*) in the short‐range region. The abscissae are normalized by the first neighboring distance. (c) NP size dependence of first coordination number. The red and blue broken lines are guides to the eye. The black solid curve corresponds to the simulated coordination number of fcc/hcp NPs with similar lattice constants. (d) NP size dependence of the coordination number of the second peak shoulder. The inset demonstrates the second peak shoulder component shown in dark green. (e) Origin of the asymmetry of the second peak in *G*(*r*). The transparent red circles are point defects. Note that it is not a phonon mode but a static schematic of local free‐volume around point defects leading to second‐shell asymmetry. (f) NP size dependence of MSDs of Ru atoms obtained by the structural refinement.

Figure 5a,b show the results of the comparison of the experimental *G*(*r*) for the short‐range regions of fcc‐based and hcp‐based NPs. Note that the interatomic distance *r* is normalized by the first nearest interatomic distance *r*_1st_ to enhance the clarity of the observations. Notably, no changes in *G*(*r*) were observed in the fcc‐based NPs, except for fcc24. In fcc24, the intensity of the third peak decreases, and the intensities on both sides of the peak increase, suggesting a more hcp‐like structure (see Figure S5). This is consistent with the results of *R*_p_ in various EUC models, as shown in Figure S1. Note that, although a pure‐PVP background was subtracted, this cannot eliminate the correlation between Ru and PVP at the particle surface. However, the X‐ray form factor of carbon in PVP is significantly smaller than that of Ru and decays more rapidly with *Q*. Therefore, any correlation between Ru and PVP is expected to be negligible compared to Ru–Ru correlations and will not affect the trends discussed below.

In contrast, hcp‐based NPs exhibit a different behavior. As the NP size decreased, the intensities of the first and third peaks decreased, whereas the asymmetry of the second peak increased. The decrease in *G*(*r*_1st_) can be attributed to the changes in coordination number associated with the increasing volume fraction of the surface region. Although the fading intensity of *G*(*r*_1st_) is more significant for hcp‐based NPs than for fcc‐based NPs, it is presumed that this difference is accompanied by the generation of atomic vacancies (point defects).

It is possible to extract the components in the PDF peaks by using the pair function, which is the Fourier transform of the structure factor of specific atomic pairs, *S_ij_* (*Q*). It can be calculated as follows:

(6) $$S_{ij}\;\left(Q\right)=1+\left(2-\delta_{ij}\right)\frac{c_{i}N_{ij}f_{i}\left(Q\right)f_{j}\left(Q\right)}{{\left|\langle f\left(Q\right)\rangle \right|}^{2}}\exp\left(-\frac{1}{2}\sigma_{ij}Q^{2}\right)\frac{\sin r_{ij}Q}{r_{ij}Q}$$

where *c_i_* is the *i*‐th mole fraction, *N_ij_* is the coordination number of *j*‐th atoms around *i*‐th atoms, *σ_ij_* is the mean square displacement, and *r_ij_* is the average distance. These structural parameters are refined using the experimental total correlation function, *T*(*r*) = *G*(*r*) + 4π*ρr*, where *ρ* is the average number density. The results of the fitting by the pair function are summarized in Figure S6. Figure 5c shows the NP size dependence of the first neighbor coordination numbers in Ru NPs evaluated by the pair function components. As expected from Figure 5a,b, the coordination number of hcp‐based NPs is significantly smaller than that of fcc‐based NPs. This difference strongly suggests the existence of point defects in hcp‐based NPs. To elucidate the existence of point defects in hcp‐based NPs, the average coordination number was simulated for NPs as a function of particle size. The coordination number of atoms constituting the fcc/hcp structure is typically 12; however, the atoms located near the NP surface exhibit smaller coordination numbers. Since the volume ratio of the central to surface regions depends on the NP size, the average coordination number decreases with decreasing NP size, as illustrated by the black solid line in Figure 5c. The variation in the first coordination number for fcc‐based NPs corresponds to the particle size dependence of the simulated average coordination number. This result suggests that fcc‐based NPs exhibit a limited number of point defects in their crystal structure. Conversely, the first coordination number for hcp‐based NPs exhibited a relatively significant variation, attributed to the NP size. This result indicates an increase in point defect density as the NP size decreases in hcp‐based NPs.

The presence of point defects affects the atomic arrangement at the second coordination shell. Figure 5d shows the coordination number of the asymmetric component of *G*(*r*) obtained using the pair‐function analysis, which increases markedly with decreasing NP size in hcp‐based samples. Figure 5e is a schematic of static local coordination loss associated with point defects, as well as the accompanying increase in local free volume. This allows for small quasi‐static displacements from average lattice sites. As a result, second‐neighbor PDF features may exhibit asymmetry and excess broadening beyond thermal effects, whereas the first–neighbor distance remains comparatively constrained by short‐range repulsion.

Figure S7 compares the nearest‐neighbour distance obtained from the average structure (from the Bragg peak) with that obtained from the local structure (from the PDF). A larger mismatch between these two metrics indicates stronger local distortion. In hcp‐based NPs, the PDF‐derived nearest‐neighbour distance is longer than the value inferred from the average lattice, implying deviations of atomic positions from their average sites. As both fcc and hcp are close‐packed, this behaviour is consistent with the local configurational free space introduced by vacancies. While vacancies can drive an overall lattice contraction (see Figure 3b,c), they can simultaneously permit short‐range relaxations that shift the first‐shell PDF peak.

Figure 5f shows the mean square displacement (MSD), corresponding to the thermal vibrations of Ru atoms, obtained by crystal PDF full‐space refinement. In the refinement, the MSD significantly contributes to the refinement in the short‐range region, *r* < 20 Å. Considering the refinement range of *G*(*r*) in this study (2.0–60 Å), the MSD reflects the strength of local atomic pair correlations rather than the average thermal vibration of the crystal. Notably, the MSD of fcc‐based NPs was approximately 50% larger than that of hcp‐based NPs. The higher MSD for fcc‐based NPs is consistent with the result of Rietveld analysis in the previous study by Song et al. [18]. This finding suggests that Ru atoms in hcp‐based NPs exhibit a strong correlation with their nearest neighbors and are bound to adjacent atoms. The origin of this strong correlation between adjacent atoms may be attributed to the coordination number reduction or the stability of the hcp structure.

It has been reported that the catalytic activities of the fcc‐ and hcp‐based Ru NPs exhibit varying dependence on NP size [9]. The catalytic activity of hcp‐based NPs increases rapidly with decreasing size below 3 nm. In contrast, fcc‐based NPs exhibit high catalytic activity, although the activity decreases at the smallest size, fcc24. Generally, it seems unreasonable that the catalytic activity of fcc24 decreases with decreasing NP size because of the increased volume fraction of the surface area. As shown in Figure 3, only the fcc24 NP exhibits a fcc layer fraction of 3/6 within the fcc‐based NP. These results suggest that the atomic arrangements formed with an fcc layer fraction of 0.5 exhibit low catalytic activity. As previously mentioned, only the lattice constant of fcc24 deviates from the others in fcc‐based NPs. Therefore, when the fcc/hcp layer fractions are equal, it is possible that atomic arrangements with distinct characteristics are formed. Notably, fcc35, fcc39, and fcc54 exhibit high catalytic activity, suggesting that the fcc layer accelerates catalytic reactions on Ru NPs. As a result, it has been elucidated that even NPs composed of a single element exhibit different electronic states and reaction active sites depending on the stacking pattern. It is expected that understanding the details of electronic states through ab initio calculations will lead to insights into catalytic reactions in other NPs and the development of novel catalysts. On the other hand, a careful investigation is required to confirm the accuracy of the electronic structure obtained by ab initio calculations. Note that a misunderstanding of the electronic structure contributing to catalytic activity may occur unless the true atomic configuration is clarified through detailed structural analysis, such as considering stacking faults. In this study, the existence of point defects in hcp‐based NPs was also demonstrated. Point defects can be detected by analyzing short‐range order based on the PDF, as they are localized disorders. This study successfully confirmed the existence of point defects in Ru NPs for the first time. Unfortunately, no correlation was observed between the point defect density and the catalytic activity of hcp‐based NPs in this study. However, the methodology for evaluating point defects in NPs using the PDF, as discussed herein, is expected to accelerate our understanding of defects in the crystal structure of NPs and their correlation with catalytic activity. Large‐scale ab initio calculations may be widely performed to elucidate the effects of point defects on the electronic structure in the future. Comparing the experimental findings of this study with simulation findings will further enhance our understanding of NP catalysts.

A PDF, obtained by analyzing high‐energy X‐ray diffraction measurements, provides structural information with high real‐space resolution. Notably, the PDF contains structural information not only from Bragg peak profiles but also from diffuse scattering components. Consequently, the PDF is particularly effective for the structural analysis of NP crystals, where periodicity is constrained by size. crystal PDF full‐space refinement successfully described stacking faults in Ru NPs using the EUC model. Moreover, the discrepancy between the experimental and calculated PDFs in the short‐range region can be simply interpreted as being due to other defects not described by the EUC model. Therefore, PDF‐based structural analysis has successfully visually demonstrated both stacking faults, which affect the average structure, and point defects, which affect the local structure, simultaneously. Structural analysis across the entire space enables the avoidance of structural inconsistencies, such as the loss of average structural information caused by refinement restricted to short‐range regions. Whole‐space crystal structure analysis using the PDF is expected to visually demonstrate defects in various materials, as well as in NPs, in the future. Although the extracted descriptors (e.g., stacking fraction, size, and defect signatures) are catalytically relevant, their impact on turnover requires kinetic measurements under reaction conditions. Accordingly, the present inferences should be regarded as qualitative hypotheses to be tested in future studies.

## 5 Summary

In this study, X‐ray total scattering measurements were conducted on fcc‐based and hcp‐based Ru NPs to obtain their *G*(*r*) values. Subsequently, crystal structural analysis was performed on fcc‐based and hcp‐based Ru NPs by crystal PDF full‐space refinement using an EUC model. It was observed that the agreement between the experimental and calculated *G*(*r*) value was strongly dependent on the fcc layer fraction in the EUC model. For fcc‐based NPs, the fcc layer fraction of 3/6 was most likely for fcc24, whereas 4/6 was observed for NPs with a size of 3 nm or larger. Notably, the lattice constant of fcc24 deviated from the trends observed in other fcc‐based NPs. Under the conditions of a fcc layer fraction = 0.5, it is possible that atomic arrangements exhibiting features different from those of other fcc‐based NPs are formed. Furthermore, considering the low catalytic activity of fcc24 compared with other fcc‐based NPs, it is presumed that the atomic arrangement observed in fcc24 is a structure with low activity. In contrast, the fcc layer fraction for hcp‐based NPs was 1/6–2/6 regardless of the NP size, indicating a structure similar to the hcp structure. Since the *R*_p_ of fcc‐based NPs was markedly small, the structure, including stacking faults, could be adequately described by the EUC model. Identifying the reasons for the low catalytic activity of fcc24 should wait for evaluation based on ab initio calculations. It is expected that the insights into the atomic arrangement based on the stacking fault density determined in this study will be helpful for the analysis. On the other hand, the high *R*_p_ values of all the hcp‐based NPs suggest that a large number of defects, such as atomic vacancies, were contained in the NPs. The asymmetry of the second peak in *G*(*r*) was only observed for the hcp‐based NPs, which supported the existence of atomic vacancies. This asymmetry corresponds to the close second‐neighbor atoms relative to the standard position due to the presence of atomic vacancies. A rigorous correlation between the defect metrics reported here and catalytic performance will require targeted activity measurements; the present work delivers the structural basis and methodology to enable such analyses.

## Conflicts of Interest

The authors declare no conflict of interest.

## Supporting information




**Supporting File 1**: smtd70600‐sup‐0001‐SuppMat.docx.

**Supporting File 2**: smtd70600‐sup‐0002‐Data.zip.

## Data Availability

Processed datasets (S(Q), G(r)), EUC model files, and key parameters are provided in the Supporting Information. Due to file size constraints, raw detector data are available from the corresponding author upon reasonable request.
