# Supplementary material for: Quantitative Analysis of Size‐Dependent Structural Disorder in Ruthenium Nanoparticles by Crystal PDF Full‐Space Refinement
Source: Small Methods. 2026 Apr 20;10(14):e01982. doi: 10.1002/smtd.202501982 (PMC13397271; doi:10.1002/smtd.202501982)
Supplement: Supplementary file 1 — Supporting File 1: smtd70600‐sup‐0001‐SuppMat.docx. [file SMTD-10-e01982-s002.docx]

Supplemental information of

Quantitative Analysis of Size-Dependent Structural Disorder in Ruthenium Nanoparticles by Crystal PDF Full-Space Refinement

Table S1 Characterizing parameters of EUC models considered to this study. See section 3.2 of the main text and Figure 1 for the meaning of variables.


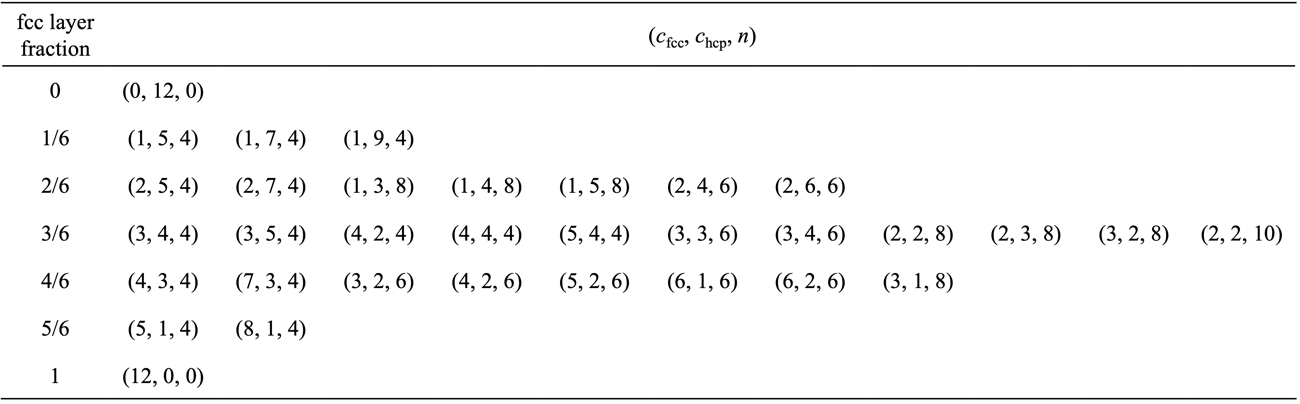


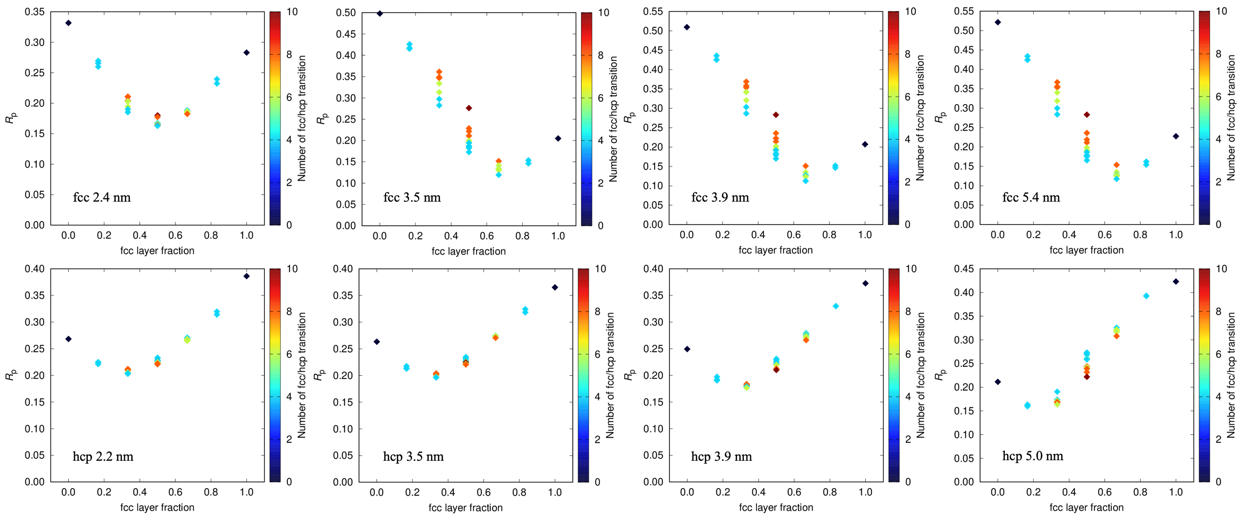


Figure S1 Fcc layer stacking dependence of *R*_p_ calculated by PDF-based structural refinement. The upper panels show the results for fcc and the lower panels are hcp. The color indicates the number of fcc/hcp transition, *n* in the EUC model for fcc and hcp nanoparticles.


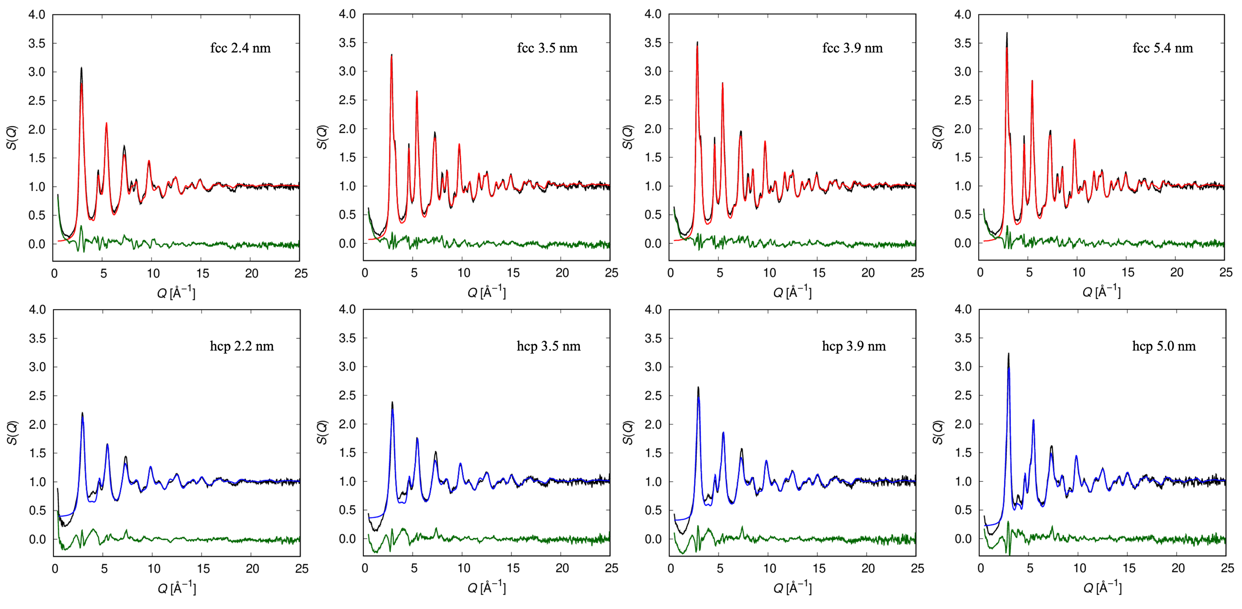


Figure S2 Comparison between experimental (black) and calculated (red for fcc, and blue for hcp nanoparticles) *S*(*Q*). The green curves correspond to the difference between them.


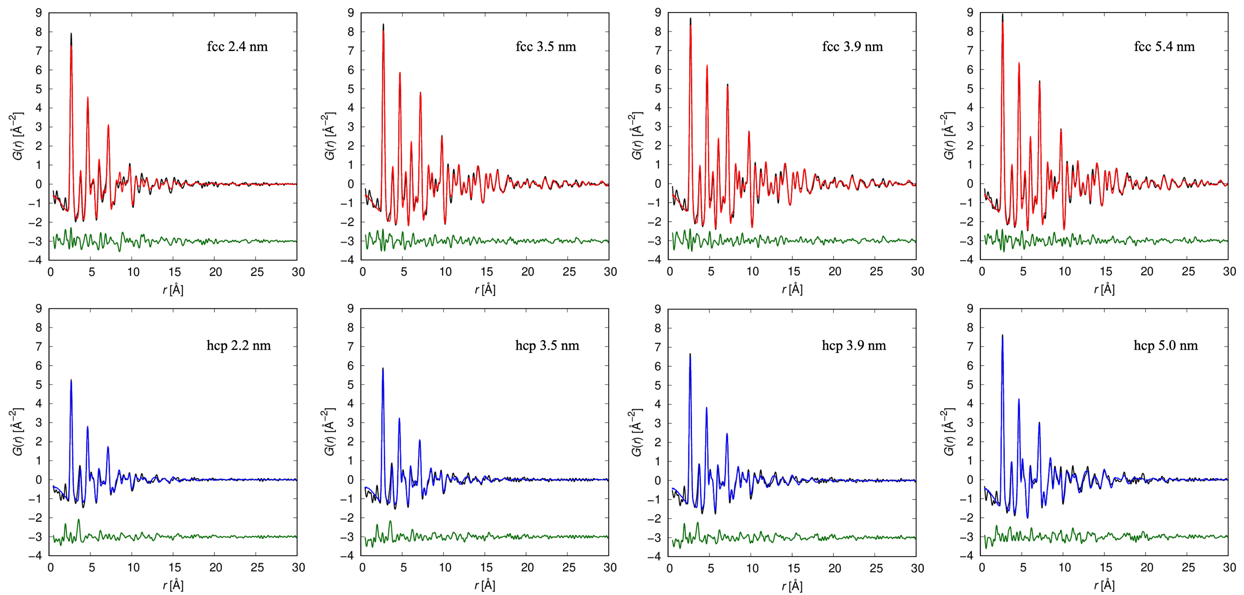


Figure S3 Comparison between experimental (black) and calculated (red for fcc, and blue for hcp nanoparticles) *G*(*r*). The green curves show the difference between them.

Table S2 Structural parameters obtained by crystal PDF full-space refinement.

| Sample | fcc24 | fcc35 | fcc39 | fcc54 |
| --- | --- | --- | --- | --- |
| Average diameter [Å] | 24 | 35 | 39 | 54 |
| Crystallite size [Å] | 19.1 | 31.4 | 33.0 | 31.5 |
| Distortion *Δx*/*x* | 0.00151 | 0.00363 | 0.00367 | 0.00101 |
| Lattice constant *a* [Å] | 2.7296 | 2.7224 | 2.7224 | 2.7208 |
| Lattice constant *c* [Å] | 26.1837 | 26.4413 | 26.3831 | 26.3188 |
| *R*_p_(2.0-60 Å) | 16.29% | 11.94% | 11.27% | 11.76% |
| Fcc layer fraction | 0.50 | 0.67 | 0.67 | 0.67 |
| Mean square displacement [Å] | 0.00636 | 0.00508 | 0.00525 | 0.00536 |
| Pseudo-voigt mixing parameter | 0.640 | 0.867 | 0.834 | 0.829 |
| Energy width *ΔE*/*E* | 0.01462 | 0.01279 | 0.01125 | 0.01190 |

| Sample | hcp22 | hcp35 | hcp39 | hcp50 |
| --- | --- | --- | --- | --- |
| Average diameter [Å] | 22 | 35 | 39 | 50 |
| Crystallite size [Å] | 18.1 | 18.9 | 20.8 | 25.1 |
| Distortion | 0.00592 | 0.00482 | 0.00633 | 0.00862 |
| Lattice constant *a* [Å] | 2.7083 | 2.7076 | 2.7063 | 2.7025 |
| Lattice constant *c* [Å] | 25.8902 | 25.8827 | 25.8774 | 25.9392 |
| *R*_p_(2.0-60 Å) | 20.29% | 19.62% | 17.67% | 15.96% |
| Fcc layer fraction | 0.33 | 0.33 | 0.33 | 0.17 |
| Mean square displacement [Å] | 0.00390 | 0.00352 | 0.00350 | 0.00336 |
| Pseudo-voigt mixing parameter | 0.653 | 0.639 | 0.532 | 0.638 |
| Energy width *ΔE*/*E* | 0.01719 | 0.01729 | 0.01634 | 0.01365 |


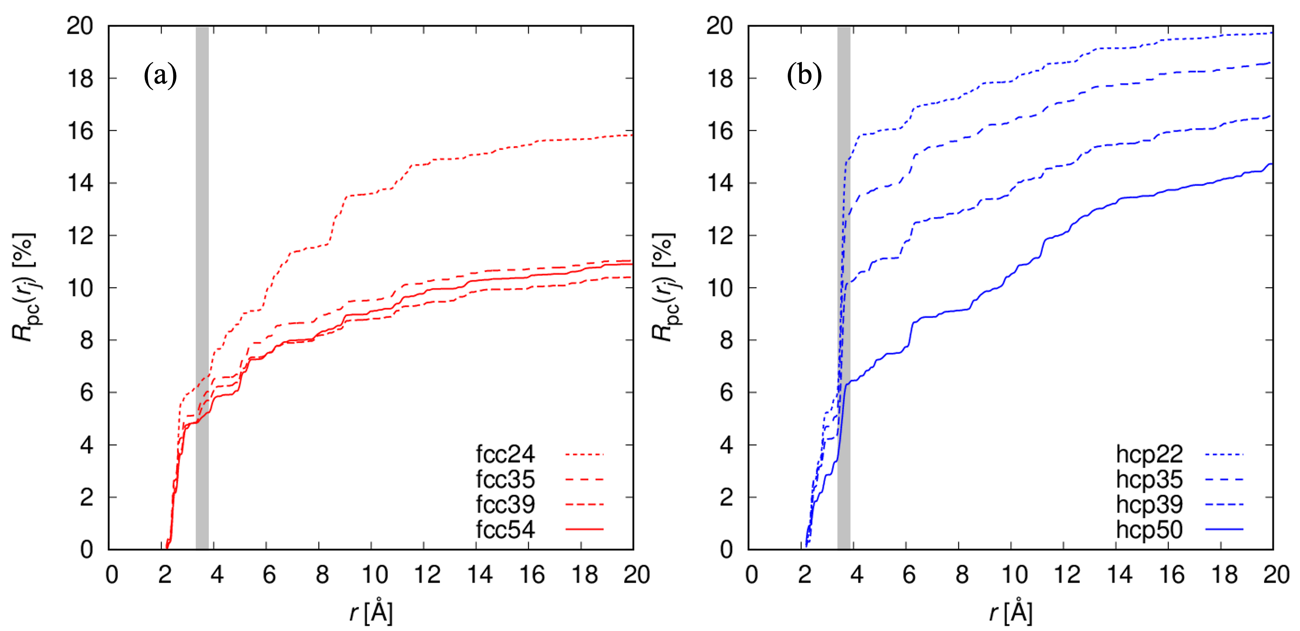


Figure S4 Comparison of cumulative *R*_p_ depending on *r*, *R*_pc_(*r*) defined as
$R_{\text{pc}}\left( r_{j} \right)=\sqrt{{\sum_{k}^{j} \left\{ G^{\text{exp}}\left( r_{k} \right)-G^{\text{calc}}\left( r_{k} \right) \right\}^{2}}/{\sum_{k}^{r=60} \left\{ G^{\text{exp}}\left( r_{k} \right) \right\}^{2}}}$. The bin size, *Δr* is 0.01 Å. The second peak position in the PDF is emphasized by the dark area.


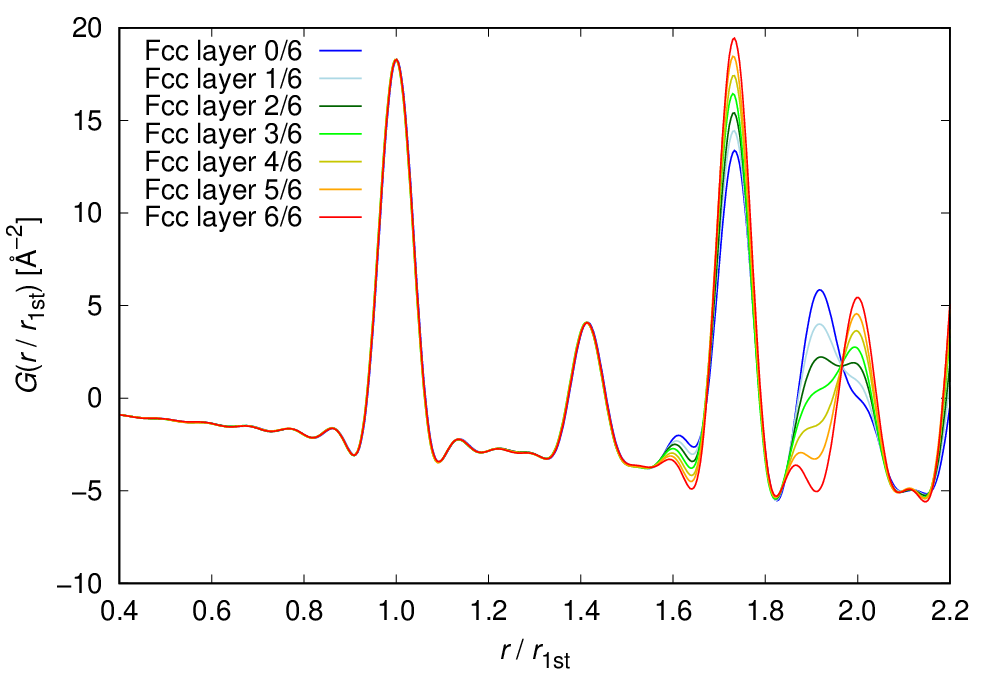


Figure S5 Variety of ideal *G*(*r*) for EUC models. The EUC models shown in the far left of the Table S1 were used in this simulation.


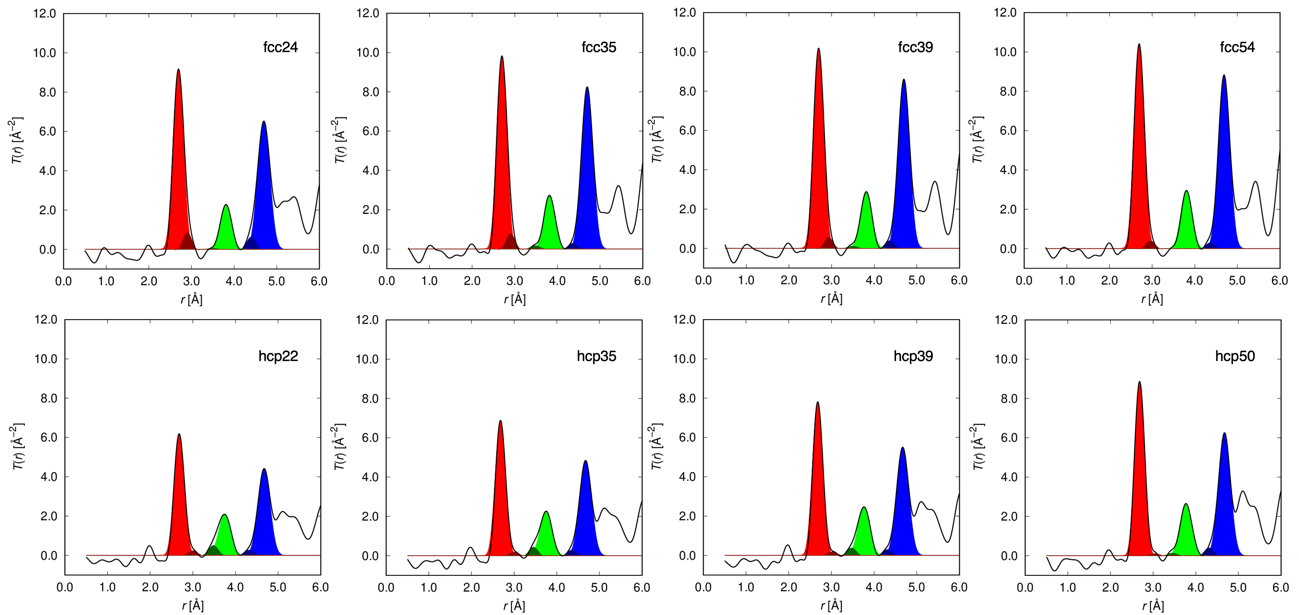


Figure S6 The correlation components in the total correlation functions, *T*(*r*). The black curves indicate the experimental *T*(*r*). The red, green, and blue area correspond to the first, second, and third peaks through the pair function by eq. (6), respectively. Brighter colors indicate the main components of each peak.


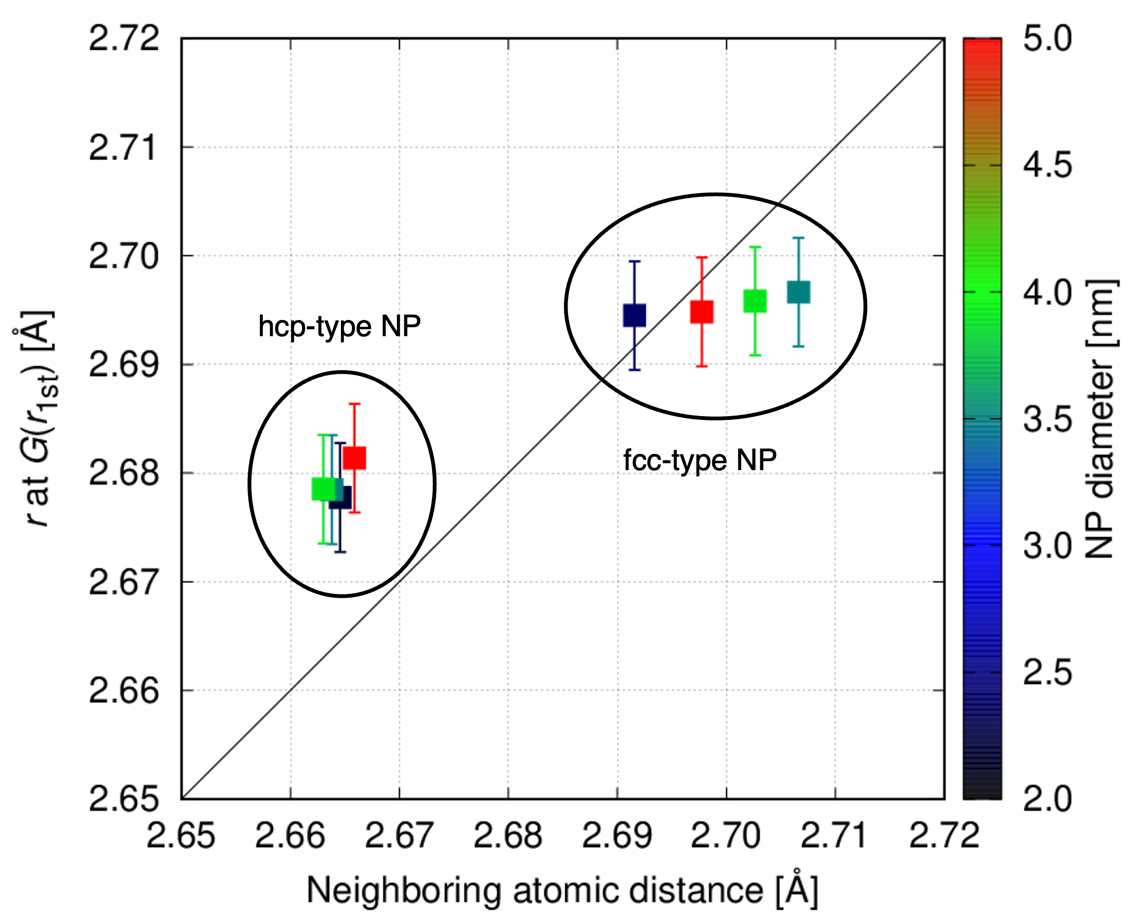


Figure S7 A comparison of neighboring atomic distance in the refined crystal structures and the first peak position of *G*(*r*).

Table S3 Calculation conditions of single-point finite-cluster Debye simulations (for Fig. S8-S12).

| Variables | values |
| --- | --- |
| Stacking | Fcc, hcp, EUC (fcc layer fraction = 0.33) |
| Particle size (single calculation) [nm] | 2.0, 3.0, 4.0, 5.0 |
| Shapes | Rod, sphere, disk |
| Aspect ratio | 0.6, 0.8, 1.0, 1.4, 1.8 |
| Vacancy fraction | 0%, 2%, 4%, 6%, 8% |

Table S4 Calculation conditions of log-normal size–distribution Debye simulations (for Fig. S13).

| Variables | values |
| --- | --- |
| Stacking | Hcp |
| Median of particle size [nm] | 2.0, 3.0, 4.0, 5.0 |
| Number of samples, *N* | 300 |
| *σ* | 0.25 |
| Shape | Sphere |
| Vacancy fraction | 0% |


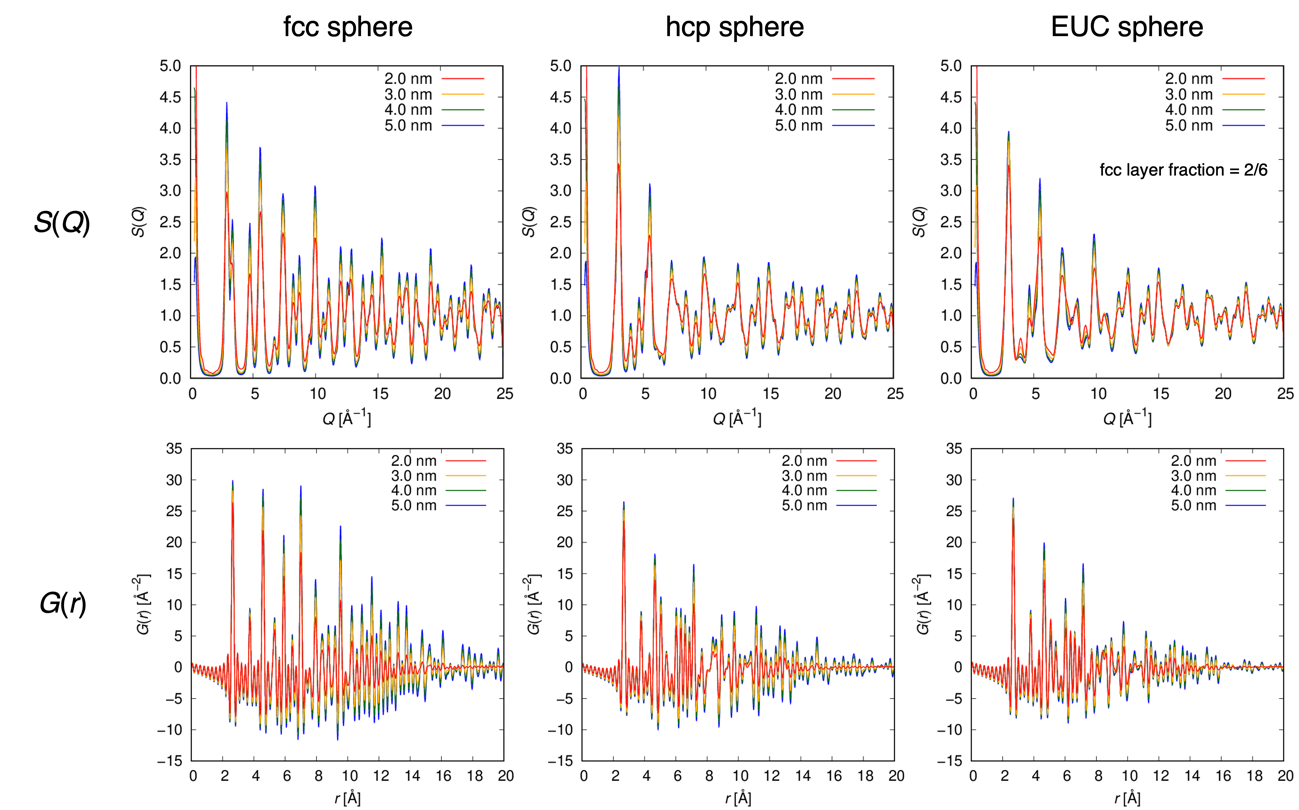


Figure S8 Calculated *S*(*Q*) (top) and *G*(*r*) (bottom) for Ru NPs using fcc, hcp, and EUC models at diameters 2–5 nm (colors) by using Debye equation.


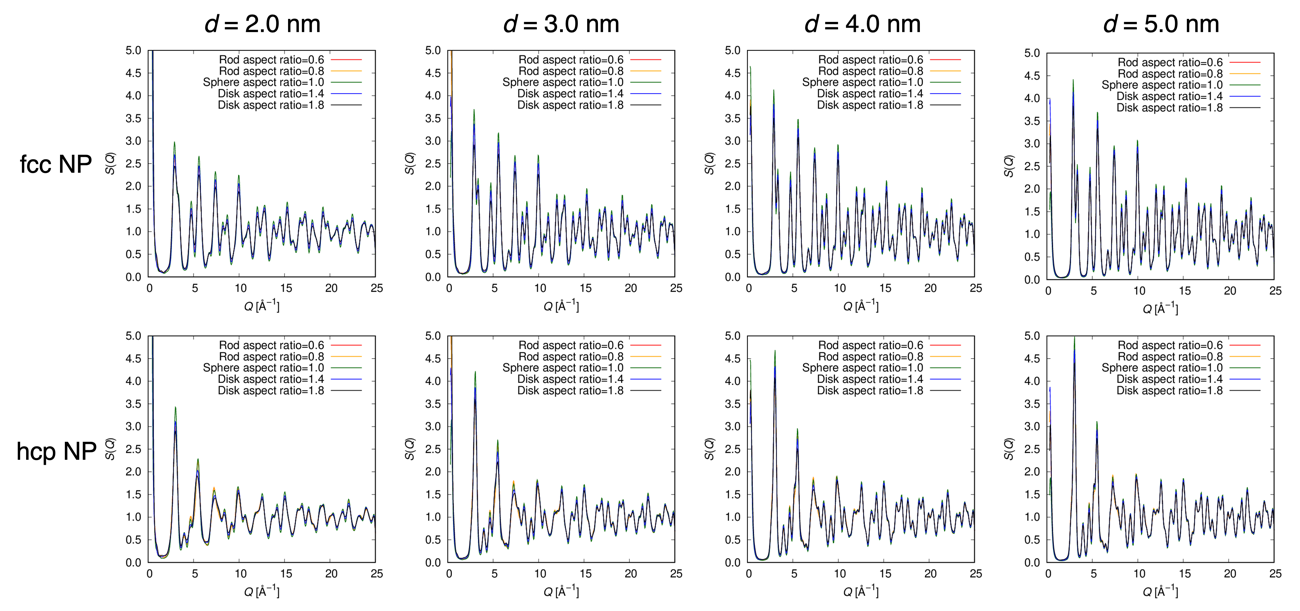


Figure S9 Aspect ratio dependence of calculated *S*(*Q*) by Debye equation. The *D* indicates the diameter of major axis in the particle.


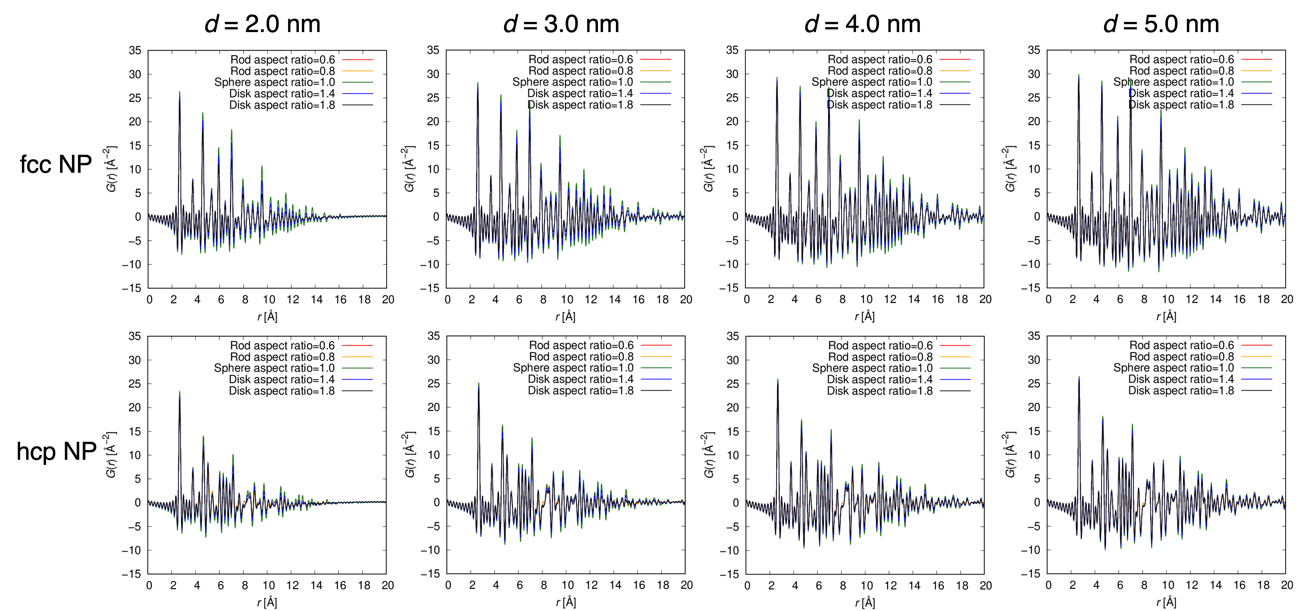


Figure S10 Aspect ratio dependence of calculated *G*(*r*) by Debye equation. The *D* indicates the diameter of major axis in the particle.


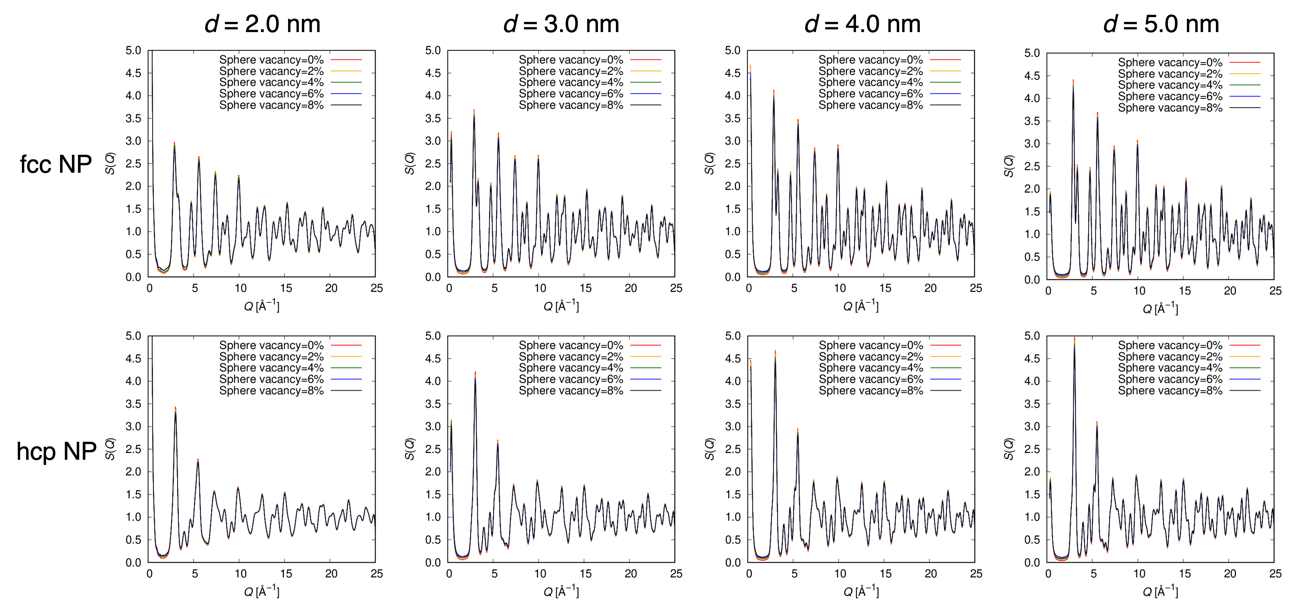


Figure S11 Vacancy fraction dependence of calculated *S*(*Q*) by Debye equation. The position of vacancies is selected using random number. The particles are sphere shape.


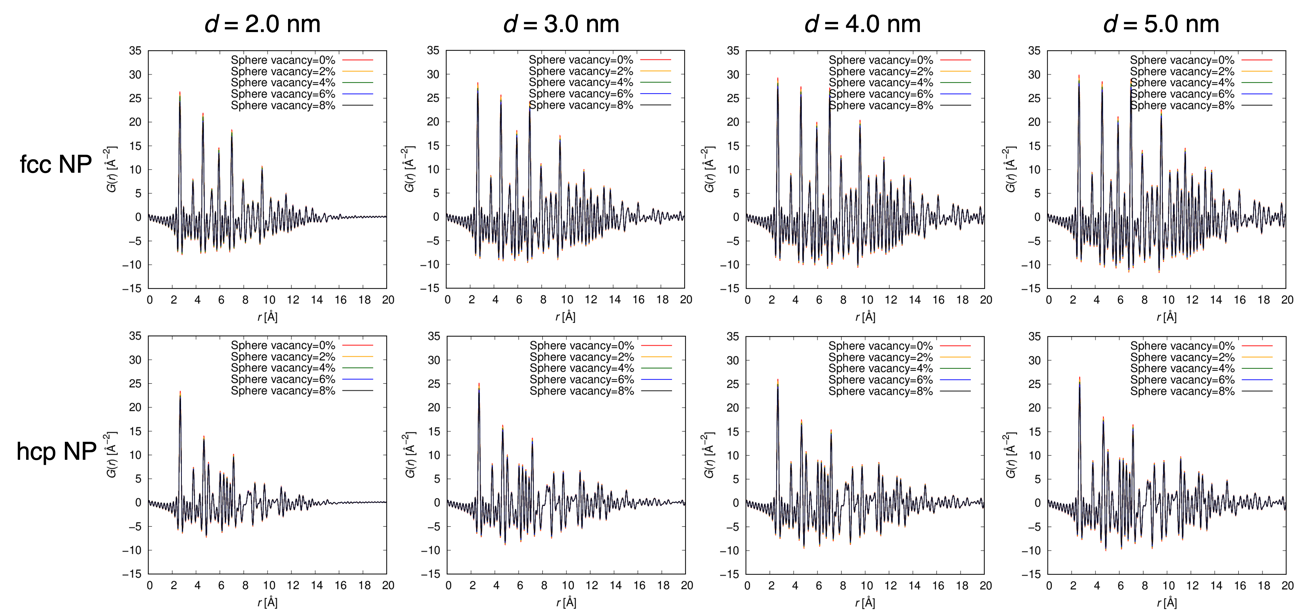


Figure S12 Vacancy fraction dependence of calculated *G*(*r*) by Debye equation. The position of vacancies is selected using random number. The particles are sphere shape.


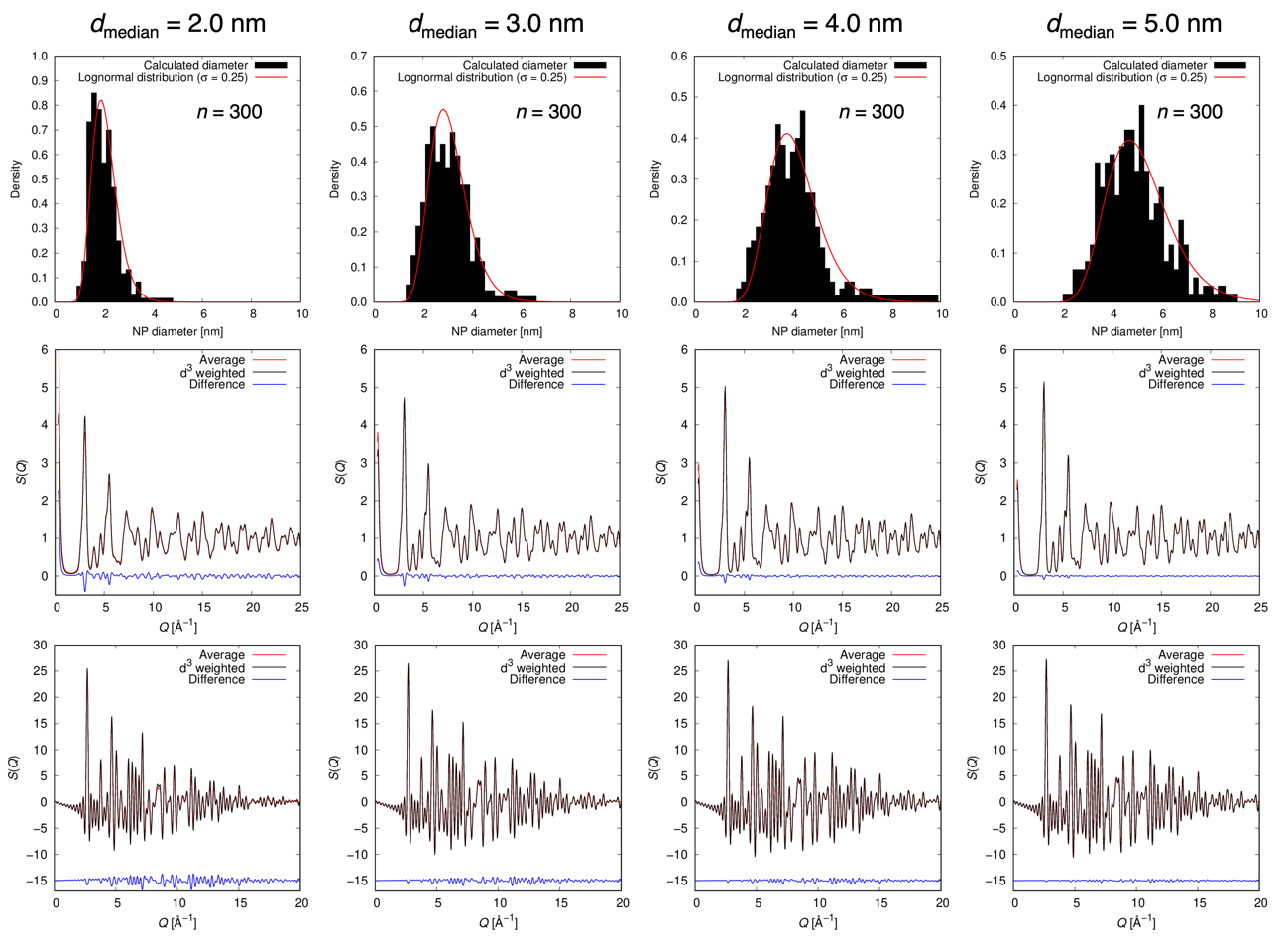


Figure S13 Effect of *d*^3^ (volume) weighting on the simulated scattering from lognormal diameter distributions. Top row: target diameter distributions with medians fixed at 2, 3, 4, and 5 nm with *σ* = 0.25, respectively. The red curve is the lognormal probability density used for sampling. The black histogram is the set of diameters actually drawn and used in the calculations (same total number of samples, 300). Middle row: *S*(*Q*) obtained by number-average over particles (red) and volume-weighted average with weights proportional to *d*^3^ (black). Bottom row: *G*(*r*) obtained by number-average over particles (red) and volume-weighted average with weights proportional to *d*^3^ (black).
